# Awareness of Microbiological Safety in Playgrounds Amid Rising Antibiotic Resistance

**DOI:** 10.1111/1758-2229.70241

**Published:** 2025-11-24

**Authors:** Rafał Łopucki, Marcin Skowronek, Anna Bilokinna, Guillermo Martinez‐de‐Tejada, Ilona Sadok

**Affiliations:** ^1^ Department of Biomedicine and Environmental Research Institute of Biological Sciences, Faculty of Medicine, Collegium Medicum, The John Paul II Catholic University of Lublin Lublin Poland; ^2^ Biotechnology Scientific Club of the John Paul II Catholic University of Lublin, Faculty of Medicine, The John Paul II Catholic University of Lublin Lublin Poland; ^3^ Department of Microbiology and Parasitology, IdiSNA (Navarra Institute for Health Research) University of Navarra Pamplona Spain; ^4^ Department of Biomedical and Analytical Chemistry Institute of Biological Sciences, Faculty of Medicine, Collegium Medicum, The John Paul II Catholic University of Lublin Lublin Poland

**Keywords:** antibiotics, bacteria, children safety, *Escherichia coli*, sandboxes

## Abstract

Playgrounds are a common feature in modern cities. Although guidelines addressing safety requirements for playground equipment have been established worldwide, none include recommendations concerning microbiological safety. Given the potential public health implications, there is a growing need to develop strategies for mitigating the risk of exposure to antibiotic‐resistant pathogens in playgrounds. The objective of this paper is to present the current state of knowledge through a systematic review of the literature, regarding microbiological safety in urban playgrounds, including an overview of the most commonly used research methodologies, the types of pathogens identified, the extent of antimicrobial resistance, and geographic differences. The review revealed significant gaps in knowledge on this topic: targeted empirical studies have been conducted relatively infrequently and only in a few countries worldwide. Even less frequently has the issue of antibiotic resistance in playground‐isolated bacteria been addressed. At the same time, antibiotic‐resistant strains represent an increasingly significant global public health concern, underscoring the need to develop global strategies to better protect playgrounds from resistant pathogens. Based on the findings, we present and discuss various factors that may influence microbiological safety in playgrounds, as well as strategies that can be implemented to address this critical issue.

## Graphical abstract

Playgrounds are identified as reservoirs for antibiotic‐resistant bacteria, posing a potential public health risk. Despite the existence of safety regulations for playground equipment, microbiological safety is treated superficially. Global strategies, including hygiene practices and antibacterial materials, are needed to reduce the spread of antibiotic‐resistant bacteria on playgrounds.

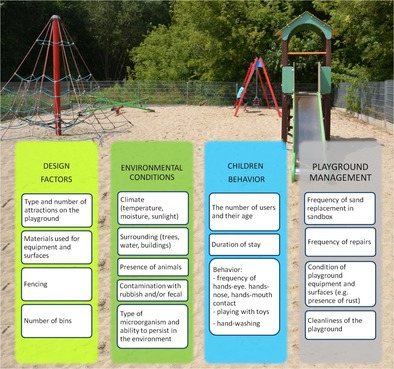

## 1 Introduction

A playground is defined as an area designed for children's play, including the site, natural features, built landscape, and any manufactured equipment and surfacing (Reedy 2024). Playgrounds are a common feature in modern cities, and their presence reflects the prioritisation of child well‐being and community welfare in urban planning and development strategies. In urban environments, playgrounds are considered essential for promoting the physical and mental health of children, providing them with a safe environment to explore and develop various skills (Ayan 2013; Błaszak and Zatoń 2015; Withagen and Caljouw 2017; Schipperijn et al. 2024). Additionally, in cities, playgrounds can serve as important community hubs, fostering social cohesion among families and neighbours. The playground area typically incorporates a variety of elements that offer a comprehensive play experience, contributing to the holistic development of children (Table 1).

**TABLE 1:** Commonly found playground equipment.

Playground component	Visual description	Trained ability
Swings	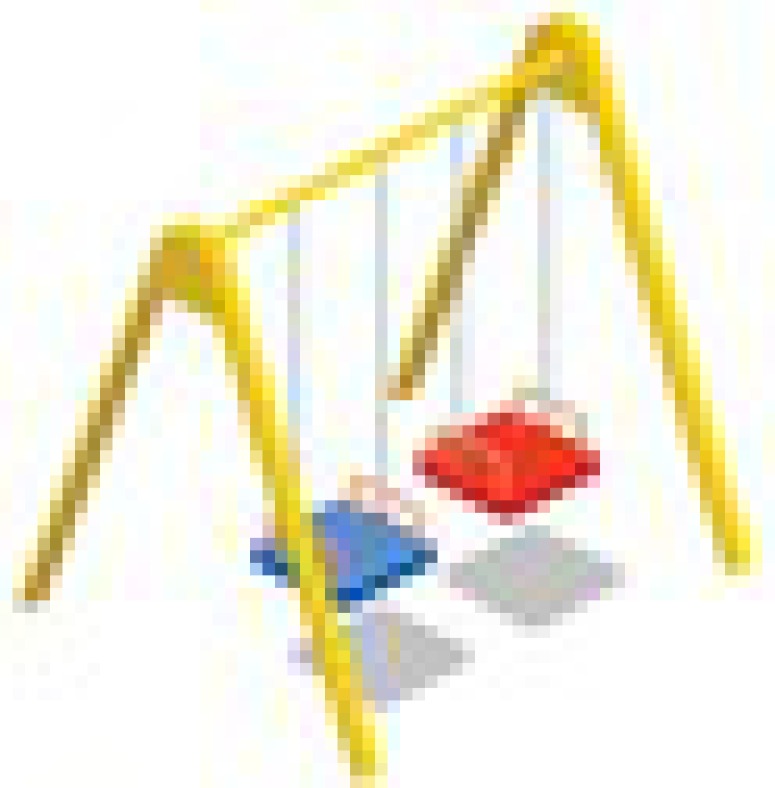	Balance, coordination, sensory stimulation through motion
Slides	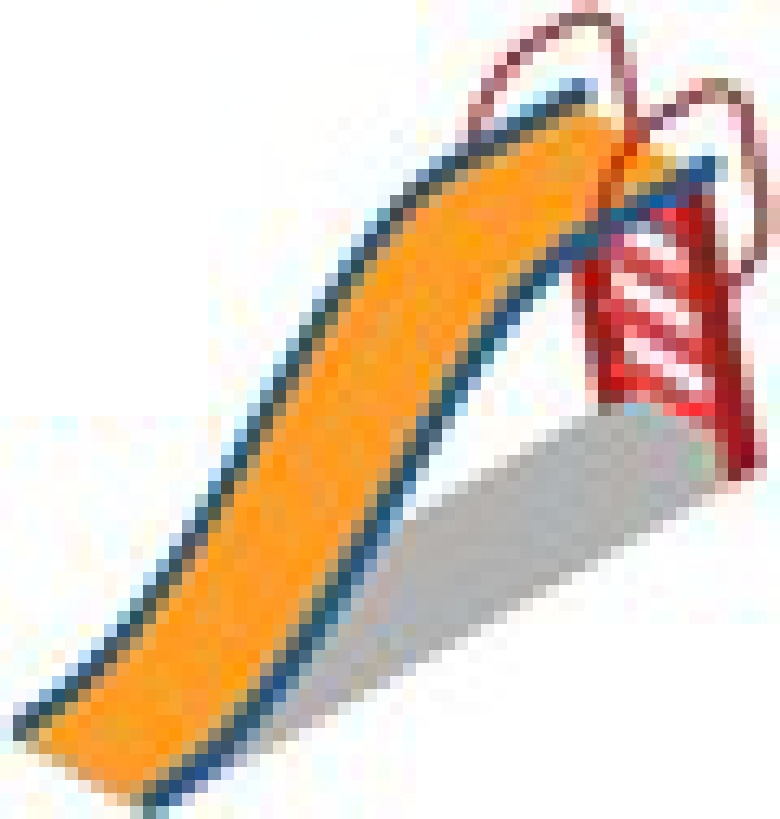	Self‐confidence, spatial awareness and coordination
Climbing structures and monkey bars	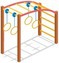	Upper body strength and coordination, problem‐solving skills, perseverance and resilience
Seesaws	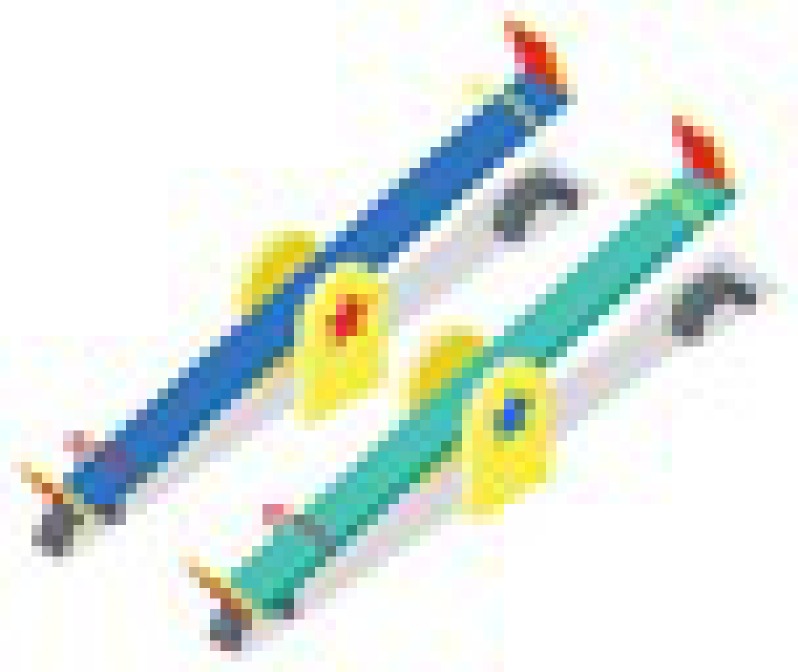	Balance, coordination and teamwork to achieve movement
Sandboxes	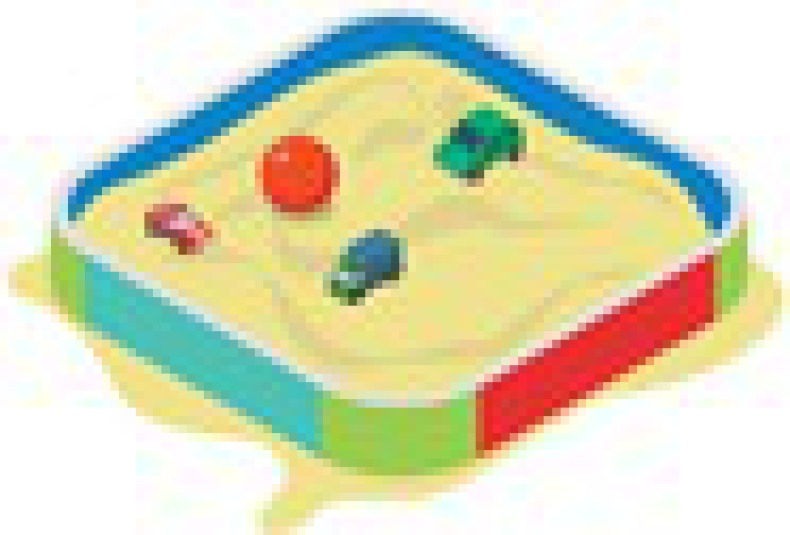	Creativity, development of fine motor skills
Playhouses and forts	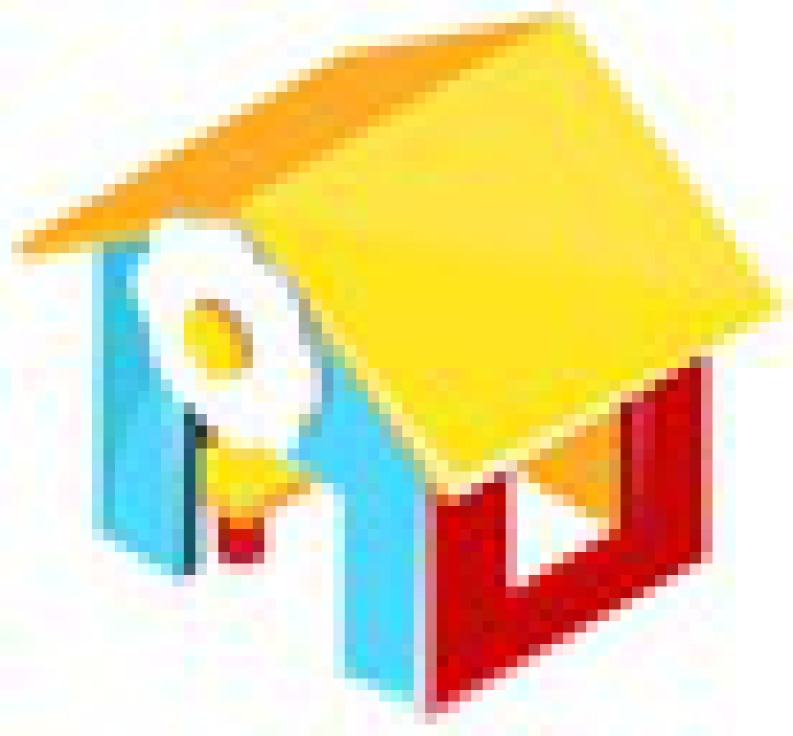	Imaginative play, social interaction, communication skills, creativity
Spinning equipment	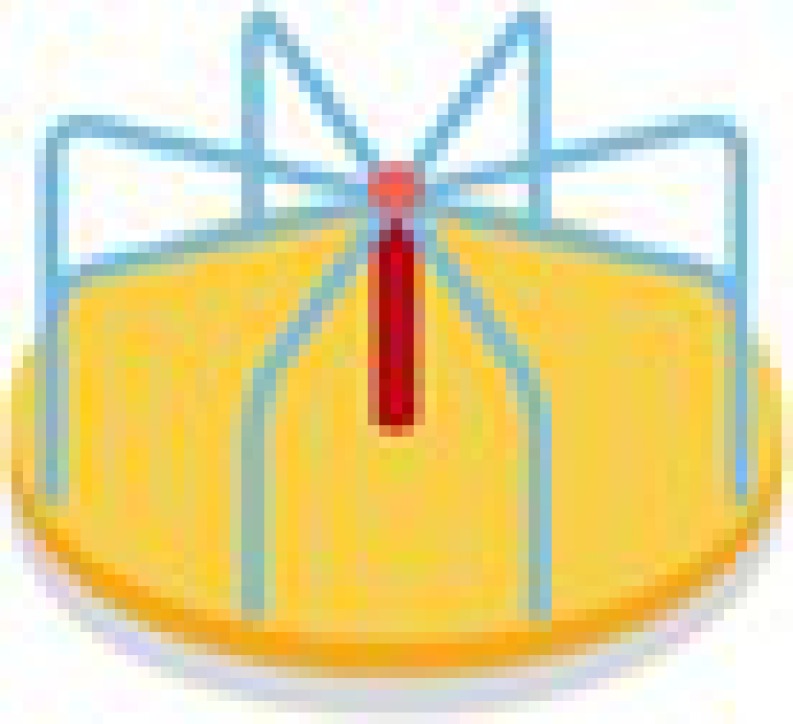	Vestibular stimulation, balance and coordination
Balancing equipment	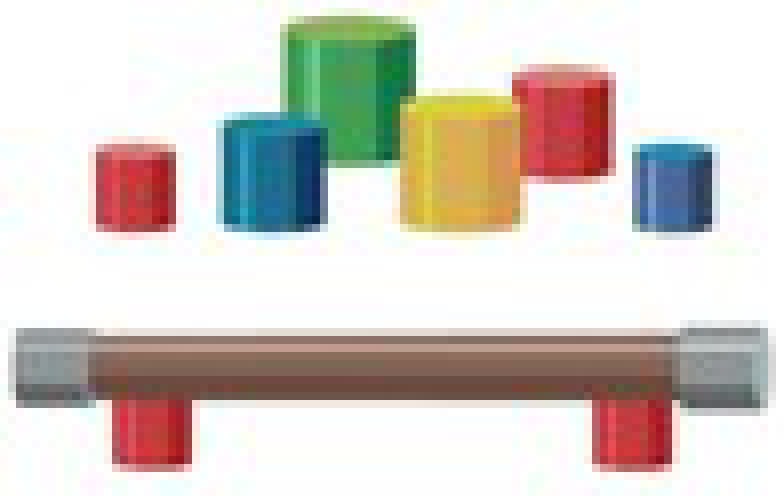	Balance, core strength, enhancement of focus and concentration
Interactive panels	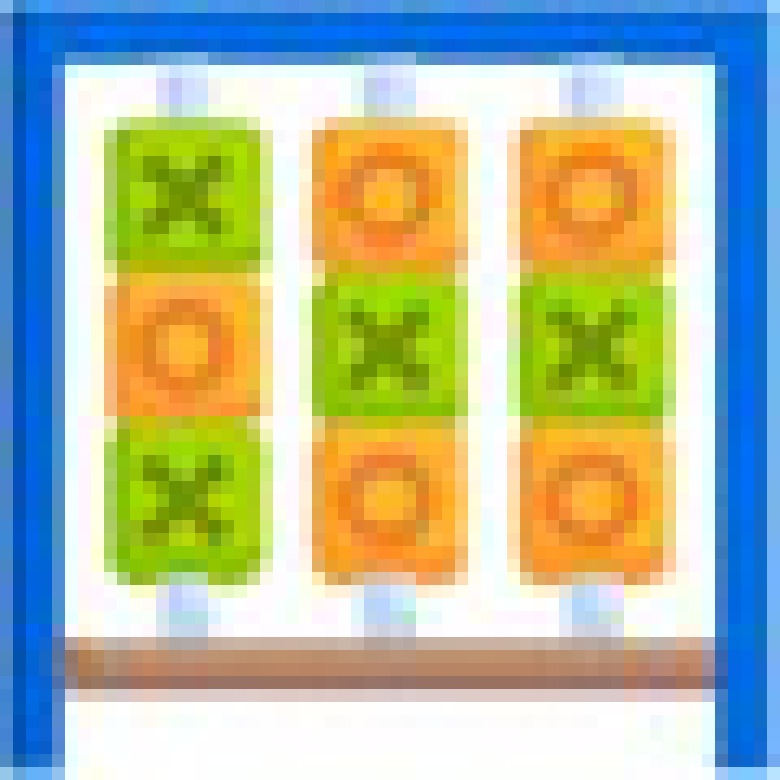	Cognitive development and problem‐solving abilities

*Note:* Images designed by Freepik (www.freepik.com).

### 1.1 Safety Requirements for Equipment and Surfacing

The diverse range of equipment found in playgrounds, combined with activities involving moving elements and elevated structures, can lead to various accidents resulting in injuries to children (Suminski et al. 2015; Tuckel and Milczarski 2018). Therefore, playground designers and managers pay special attention to safety measures to protect children. Guidelines addressing general safety requirements for playground equipment and surfacing have been established worldwide (Table 2). They cover appropriate equipment design, layout (including requirements for falling space, free space, and the impact area ‐ also known as the fall zone ‐ around the playground equipment), protection against falls and entrapment, and maintenance of protective surfacing to prevent playground‐related injuries and deaths. However, none of them include recommendations for parasitic or microbiological safety of playgrounds. Meanwhile, playgrounds, often considered safe spaces for children, can, in fact, serve as reservoirs for a variety of harmful bacteria, viruses, and fungi. These microorganisms can be transmitted through contact with contaminated surfaces, soil, water, or other children.

**TABLE 2:** Safety requirements and test methods for playground equipment and surfacing.

Region	Norm (year^a^)	Description	Reference to microbiological safety issues
European countries	EN 1176–1 (2017)	Playground equipment and surfacing ‐ General safety requirements and test methods	NO
	EN 1176 2–6 (2017–2020)	Playground equipment and surfacing ‐ Additional specific safety requirements and test methods for swings, slides, cableways, carousels, rocking equipment	
	EN 1176–7 (2020)	Playground equipment and surfacing ‐ Guidance on installation, inspection, maintenance and operation	
	EN 1176–10 (2008)	Playground equipment and surfacing ‐ Additional specific safety requirements and test methods for fully enclosed play equipment	
	EN 1176–11 (2014)	Playground equipment and surfacing Additional specific safety requirements and test methods for spatial network	
	EN 1177 (2018)	Impact attenuating playground surfacing. Methods of test for determination of impact attenuation	
Russia	GOST R 59010 (2020)	Equipment and covering for playgrounds. Additional safety requirements and test methods for universal playground devices	NO
	TR EAEU 042/2017 (2017)	The technical regulation of the Eurasian Economic Union on safety of children's playgrounds	
Australia	AS 4685.0 (2017)	Playground equipment and surfacing – Development, installation, inspection, maintenance and operation	NO
	AS 4685.1–6 (2021)	Playground equipment – General safety requirements and test methods. Additional specific requirements for swings; slides; runways; carousels; rocking equipment	
	AS 4685.11 (2012)	Playground equipment – Additional specific safety requirements and test methods for spatial network	
	AS 4422 (2016)	Playground surfacing – Specifications, requirements and test method	
United States	ASTM F1487 (2021)	Standard consumer safety performance specification for playground equipment for public use	NO
	ASTM F2373 (2011)	Standard consumer safety performance specification for public use play equipment for children 6 months through 23 months	
	ASTM F1292 (1999)	Standard specification for impact attenuation of surface systems under and around playground equipment	
	ASTM F2075 (2020)	Standard specification for engineered wood fibre for use as a playground safety surface under and around playground equipment	
	ASTM F2223 (2019)	Standard guide for ASTM standards on playground surfacing	
	ASTM F2479 (2017)	Standard guide for specification, purchase, installation and maintenance of poured‐in‐place playground surfacing	
	ASTM F1951 (2021)	Standard specification for determination of accessibility of surface systems under and around playground equipment	
United States	ASTM F1816 (2018)	Standard safety specification for drawstrings on children's upper outerwear
	NO
		ASTM F2049 (2000)	Standard guide for fences/barriers for public, commercial, and multi‐family residential use outdoor play areas
		ASTM F1148 (2022)	Standard consumer safety performance specification for home playground equipment
ASTM F1918 (2021)		Standard safety performance specification for soft contained play equipment

Abbreviation: ASTM, American Society of Testing and Materials Standards.

^a^ Year of original adoption or last revision.

### 1.2 Microbiological and Parasitological Safety

Studies have reported that health and safety in playgrounds and public spaces are also associated with the presence of pathogenic microorganisms or parasites. For instance, playgrounds, particularly sandpits, can harbour *Toxocara* spp. eggs (Düwel 1984; Otero et al. 2018; Kuśmierek et al. 2020; Tyungu et al. 2020; Ristić et al. 2020; Lorenzo‐Rebenaque et al. 2023; Matras et al. 2023). Sandpits in playgrounds may also be contaminated with *Toxoplasma gondii* (Pacheco‐Ortega et al. 2019), which can be transmitted to humans through the ingestion of soil containing sporulated oocysts derived from feline faeces. Additionally, playing in playgrounds poses a risk of contracting cryptosporidiosis (Gharpure et al. 2019). Furthermore, several fungal species potentially harmful to children have also been detected in sandpits (Kaltseis et al. 2009; Jain and Sharma 2012; Wójcik et al. 2016; Glushakova et al. 2024), while pathogenic respiratory viruses have been identified in public spaces (Ikonen et al. 2018). Various bacterial pathogens have also been found on different playground elements (e.g., Pérez et al. 2010; Badura et al. 2014; Błaszak and Zatoń 2015; Thapaliya et al. 2019; Caliskan et al. 2021; Simanjuntak et al. 2023).

**FIGURE 1:**
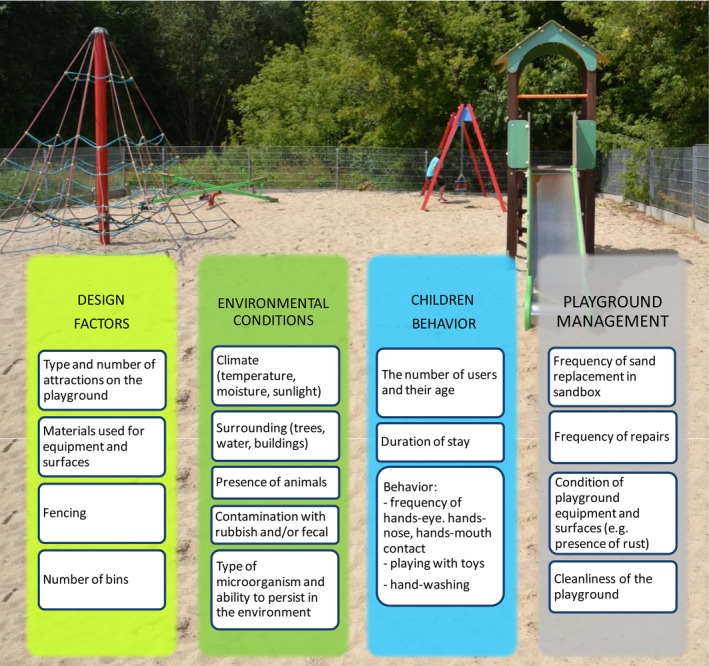
Main risk factors for children's exposure to microbial pathogens in playgrounds.

Children are particularly vulnerable to severe health consequences when exposed to the aforementioned parasites and pathogens due to their underdeveloped immune systems. The primary transmission pathways involve incidental hand‐to‐mouth, hand‐to‐nose, and hand‐to‐eye contact (Chatziprodromidou et al. 2021). Furthermore, during play, children may transfer microbial pathogens from their hands to playground equipment and vice versa (Martínez‐Bastidas et al. 2014). Examples of risk factors contributing to children's exposure to microbial pathogens in playgrounds are presented in Figure 1.

Despite these risks, the issue of microbiological safety for children on playgrounds is typically not governed by specific regulations. In most cases, the legal basis for hygiene requirements on playgrounds is derived from general regulations concerning the prevention and control of infections and communicable diseases in humans. For instance, in Poland (a member of the European Union), these matters are regulated by the Act (Dz. U. 2008 Nr 234, poz. 1570), which broadly stipulates that property owners, possessors, or managers are required to maintain their premises in proper sanitary and hygienic conditions to prevent infections and communicable diseases. Specifically, they must: (1) manage waste and sewage appropriately; (2) control rodents, insects, and pests; (3) remove dead animals from the property; and (4) clean up animal excrement. Although not explicitly stated, these regulations substantiate practices aimed at reducing pathogen transmission, such as regular sand replacement in sandboxes, cleaning of these areas, installation of fences to keep animals out of playgrounds, and inspections by the local Sanitary and Epidemiological Station.

### 1.3 Antimicrobial Resistance of Pathogenic Microorganisms as an Emerging Risk Factor

Microbiological hazards present in playgrounds have been significantly exacerbated in recent years by the spread of antimicrobial resistance among pathogenic microorganisms. Antimicrobial resistance occurs when bacteria develop mechanisms to survive exposure to drugs intended to eliminate them. The rise in antimicrobial resistance has been driven by several factors, including the overuse and misuse of antibiotics in human medicine, veterinary practice, and agriculture (Podolsky 2018; WHO 2021). This has led to the selection of resistant strains of microorganisms that can persist in the presence of drugs that would normally inhibit their growth or eradicate them, allowing these strains to persist and spread, thereby complicating their removal from the environment (Akram et al. 2023).

The presence of antibiotic‐resistant bacteria in playgrounds means that common infections could potentially lead to more severe health outcomes, including prolonged illnesses, increased treatment costs, and a higher likelihood of complications (Salam et al. 2023). Moreover, a recent study suggests that patients colonised with antibiotic‐resistant bacteria (ESBL *Klebsiella pneumoniae* and *Escherichia coli* ) may act as durable reservoirs for ongoing transmission of ESBLs, and that they are at prolonged risk of recurrent infection with colonising strains (Aguilar‐Bultet et al. 2023). However, it remains unclear whether bacteria with resistance mechanisms can survive longer than antibiotic‐sensitive strains on surfaces commonly found in playgrounds, and whether this consequently increases the risk of exposure for children.

### 1.4 Aim and Scope of the Literature Review

Given the potential public health implications, there is a growing need to develop strategies for mitigating the risk of exposure to antibiotic‐resistant pathogens in playgrounds. However, creating such strategies requires a comprehensive understanding of the issue based on scientific evidence. Therefore, the objective of this review is to present the current state of knowledge regarding microbiological safety in urban playgrounds, including an overview of the most commonly used research methodologies, the types of pathogens identified, the extent of antibiotic resistance, and geographic differences. This objective was achieved through a systematic review of the literature on microbiological studies conducted within playground areas.

Additionally, based on the general microbiology literature, we present and discuss various factors influencing microbiological safety in playgrounds, as well as strategies that can be implemented to enhance it.

## 2 Methods ‐ Literature Search and Inclusion/Exclusion Criteria

To gather literature data on microbiological research conducted in playgrounds, the PRISMA method (Page et al. 2021) was employed. This method involves the preliminary identification of articles through selected databases, screening the results obtained, assessing their eligibility, and ultimately including them in the review.

A literature search was conducted using the scientific databases Scopus and PubMed. For the initial identification of articles, the search terms “playground” (or “playgrounds”) and “bacteria” were used. No time constraints were applied to the search results. In Scopus, the search was performed using the “title‐abstract‐keywords” option, while in PubMed, the “all fields” option was used. The search was carried out in October 2023 and subsequently refined in October 2025. The search yielded 151 records in Scopus and 112 records in PubMed.

The search results from both databases were combined, and duplicates were removed. Subsequently, using the title and abstract, all entries were assessed for relevance to the topic under consideration (i.e., articles specifically addressing empirical studies on the prevalence and/or characteristics of bacteria on playgrounds). The suitability of the selected articles was then finalized based on a full‐text review. Only articles published in English and in peer‐reviewed journals were included (i.e., grey literature, dissertations, and conference papers were excluded).

Ultimately, the selected pool of articles (15 items) was analysed for the chosen topics and summarised in tables discussed later in the text (Tables 3 and 4). Specifically, attention was given to which materials or surfaces were assessed for bacterial presence, which bacterial species were investigated, how frequently bacteria were found on the examined surfaces or materials, whether the bacteria were tested for antibiotic resistance, and, if so, which antimicrobial agents were used, as well as the geographical regions in which the studies were conducted.

**TABLE 3:** Relevant epidemiological data obtained from publications on microbial safety of playgrounds.

Sample	Sampling site	Bacteria	N_total_	N_positive_ (mean concentration)	Positivity rate	Country (city)	References
Sand	Sandboxes	*Escherichia coli*	45	22 (2.6 × 10^3^ CFU/g)	48.9%	Austria (Graz)	(Badura et al. 2014)
		Coliform bacteria^a^	45	44 (3.0 × 10^4^ CFU/g)	97.8%		
Sand	Sandboxes	*Enterococci*	42	42 (111 MPN/g (d))	100%	USA (New York)	(Leri et al. 2024)
		*Escherichia coli*	42	42 (13 MPN/g (d))	100%		
Sand	Sandboxes	*Escherichia coli*	32	19	59.4%	Poland (Szczecin, Gorzów Wielkopolski)	(Błaszak and Zatoń 2015)
		Coliform bacteria	32	26	81.3%		
Sand	Artificial beaches (areas of playground for children)	*Salmonella*	18	0	—	Poland (Szczecin)	(Zatoń and Błaszak 2015)
		*Escherichia coli*	18	2	11.1%		
		Coliform bacteria^b^	18	10	55.6%		
Sand	Sandboxes	*Clostridium difficile* (current official name—*Clostridioides difficile*)	40	21	52.5%	Spain (Madrid)	(Orden et al. 2017)
Sand	Playground zone	*Escherichia coli*	140	N.a. (129.96 CFU/g (d), 6.1 × 10^3^ CFU/g (w))	N.a.	Greece (Patras and Pyrgos)	(Chatziprodromidou et al. 2021)
		*Staphylococcus aureus*	140	N.a. (0 CFU/g (d), 5.06 × 10^3^ CFU/g (w))	N.a.		
		*Pseudomonas aeruginosa*	140	N.a. (32.81 CFU/g (d), 691.781 CFU/g (w))	N.a.		
Soil (sand, gravel, loam, clay)	Playground surface (swings, slides)	*Salmonella*	79	24	30.4%	USA (Island of Guam)	(Haddock and Nocon 1986)
Soil	Playground surface	*Helicobacter pylori*	78	7	9%	Spain (Barcelona)	(Pérez et al. 2010)
Soil	Playground area	*Escherichia coli*	140	17	12.1%	Turkey (Ankara)	(Caliskan et al. 2021)
Swab	Playground equipment	*Staphylococcus aureus*	280	89	31.8%	USA (Northeast Ohio)	(Thapaliya et al. 2019)
Swab	Playground equipment	*Staphylococcus aureus*	355	10	2.81%	Hungary (16 cities)	(Horváth et al. 2024)
Swab	Slides, swings, wheels, seesaws, other toys	*Escherichia coli*	414	23	5.6%	Turkey (Ankara)	(Caliskan et al. 2021)
Swab	Children's hands	Gram‐negative bacteria^c^	160	65	40.6%	Germany (Göttingen)	(Simanjuntak et al. 2023)
			180	97	53.9%	Indonesia (Medan)	
			171	114	66.7%	Indonesia (Siberut)	
Swab	Playground slides	*Acinetobacter pittii*	39	2	5.1%	Northern Jordan	(Ababneh et al. 2022)
Wild‐bird faecal samples	Playground area	*Campylobacter* spp.	200	35	17.5%	Iran (Mashhad)	(Abdollahpour et al. 2015)
Canine faecal samples	Playground area	*Escherichia coli*	50	20	40.0%	Ecuador (Quito)	(Ortega‐Paredes et al. 2019)

^a^ *Klebsiella pneumoniae* , *Klebsiella oxytoca* , *Citrobacter freundii* , *Enterobacter amnigenus* , *Enterobacter aerogenes* , *Serratia marcescens* .

^b^ Not specified.

^c^ Enterobacterales, Pseudomonadales, and others; MPN ‐ most probable number of microorganisms; n.a. ‐ data not available; d ‐ dry season; w ‐ wet season.

**TABLE 4:** Antibiotic resistance profiles of *Escherichia coli* and *Staphylococcus aureus* isolates from playground elements and surfacing.

Agents	*Escherichia coli*			*Staphylococcus aureus*	
	Sample type: soil	Sample type: swab	Sample type: sand	Sample type: swab	Sample type: swab
	(*n* = 17)	(*n* = 23)	(*n* = 96)	(*n* = 10)	(*n* = 280)
Amikacin (AK)	R (0%) I (5.8%) S (94.2%)	R (0%) I (0%) S (100%)	R/I (0%) S (100%)	—	—
Amoxicillin/Clavulanic acid (AMC)	—	—	R/I (9.4%) S (90.6%)	—	—
Ampicillin (AMP)	R (11.7%) I (0%) S (88.3%)	R (21.8%) I (4.3%) S (73.9%)	R/I (12.5%) S (87.5%)	—	—
Aztreonam (ATM)	R (0%) I (0%) S (100%)	R (0%) I (4.3%) S (95.7%)	R/I (0%) S (100%)	—	—
Cefepime (FEP)	—	—	R/I (0%) S (100%)	—	—
Cefotaxime (CTX)	R (0%) I (0%) S (100%)	R (0%) I (4.3%) S (95.7%)	R/I (0%) S (100%)	—	—
Cefoxitin (FOX)	—	—	R/I (3.1%) S (96.9%)	R (0%) S (100%)	—
Ceftazidime (CAZ)	R (0%) I (0%) S (100%)	R (0%) I (4.3%) S (95.7%)	R/I (0%) S (100%)	—	—
Cefuroxime (CXM)	—	—	R/I (3.1%) S (96.9%)	—	—
Chloramphenicol (CHL)	—	—	R/I (2.1%) S (97.9%)	—	—
Ciprofloxacin (CIP)	R (0%) I (5.8%) S (94.2%)	R (8.7%) I (8.7%) S (82.6%)	R/I (1%) S (99%)	R (40%) S (60%)	—
Clindamycin (DA)	—	—	—	R (20%) S (80%)	—
Ertapenem (ETP)	R (0%) I (0%) S (100%)	R (0%) I (0%) S (100%)	—	—	—
Erythromycin (ERY)	—	—	—	R (20%) S (80%)	—
Fosfomycin (FOS)	—	—	R/I (0%) S (100%)	—	—
Gentamycin (CN)	R (0%) I (88.3%) S (11.7%)	R (8.7%) I (91.3%) S (0%)	R/I (0%) S (100%)	R (0%) S (100%)	—
Imipenem (IPM)	R (0%) I (0%) S (100%)	R (0%) I (0%) S (100%)	R/I (0%) S (100%)	—	—
Levofloxacin (LEV)	—	—	—	R (0%) S (100%)	—
Meropenem (MEM)	R (0%) I (0%) S (100%)	R (0%) I (0%) S (100%)	R/I (0%) S (100%)	—	—
Moxifloxacin (MXF)	—	—	R/I (2.1%) S (97.9%)	—	—
Nalidixic acid (NAL)	—	—	R/I (4.2%) S (95.8%)	—	—
Oxacillin (OX)	—	—	—	R (0%) S (100%)	R (3.9%) S (96.1%)
Penicillin (PCN)	—	—	—	R (20%) S (80%)	—
Piperacillin (PIP)	—	—	R/I (10.4%) S (89.6%)	—	—
Piperacillin/Tazobactam (TPZ)	R (0%) I (0%) S (100%)	R (0%) I (8.7%) S (91.3%)	R/I (5.2%) S (94.8%)	—	—
Streptomycin (STR)	R (17.6%) I (64.7%) S (17.6%)	R (8.7%) I (73.9%) S (17.4%)	—	—	—
Tetracycline (TET)	—	—	R/I (6.3%) S (93.7%)	R (0%) S (100%)	—
Tigecycline (TGC)	—	—	R/I (0%) S (100%)	—	—
Trimethoprim/Sulfamethoxazole (SXT)	R (5.8%) I (0%) S (94.2%)	R (21.8%) I (0%) S (78.2%)	—	—	—
Tobramycin (TOB)	R (0%) I (100%) S (0%)	R (8.7%) I (91.3%) S (0%)	—	—	
Vancomycin (VAN)	—	—	—	R (0%) S (100%)	—
Ref.	(Caliskan et al. 2021)	(Caliskan et al. 2021)	(Badura et al. 2014)	(Horváth et al. 2024)	(Thapaliya et al. 2019)
The location of the studies	Turkey	Turkey	Austria	Hungary	USA

*Note:* I ‐ intermediate susceptibility; R ‐ resistant; S ‐ sensitive.

## 3 Results ‐ Current Knowledge on Microbiological Safety of Playgrounds

The systematic literature review revealed that the topic of microbiological safety in playgrounds is rarely addressed, with only 15 relevant articles identified. The earliest studies on this topic found in the analysed databases date back to 1986, while the most recent are from 2025.

**FIGURE 2:**
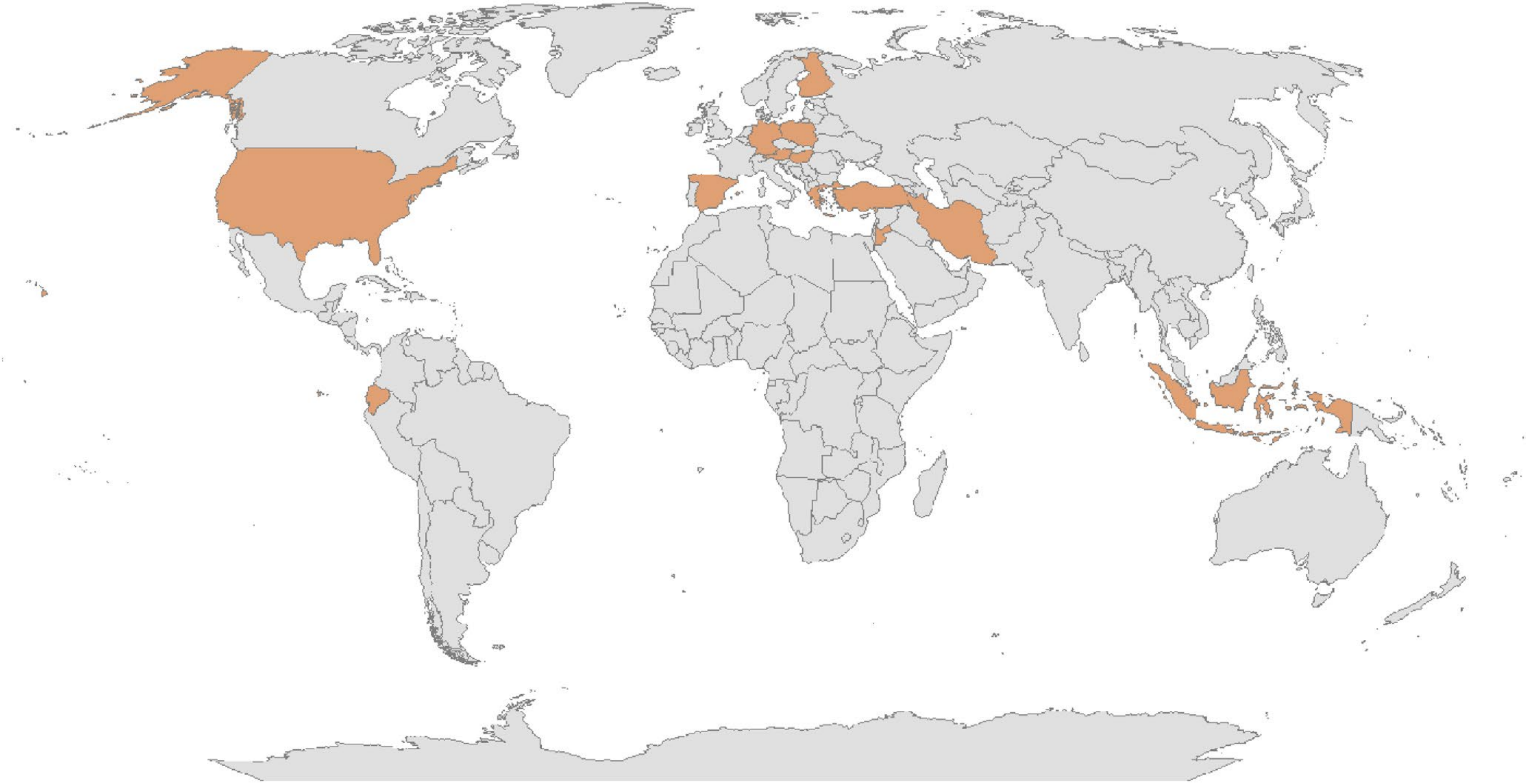
Global distribution of countries with published data on playground‐associated bacteria.

Geographic distribution of research sites worldwide is presented in Figure 2. As shown, studies have been conducted only in certain countries in Europe, Asia, North America, and South America. Data from many regions of the world are lacking, highlighting the significant need for a more comprehensive global assessment of this phenomenon.

### 3.1 Types of Samples Analysed

**FIGURE 3:**
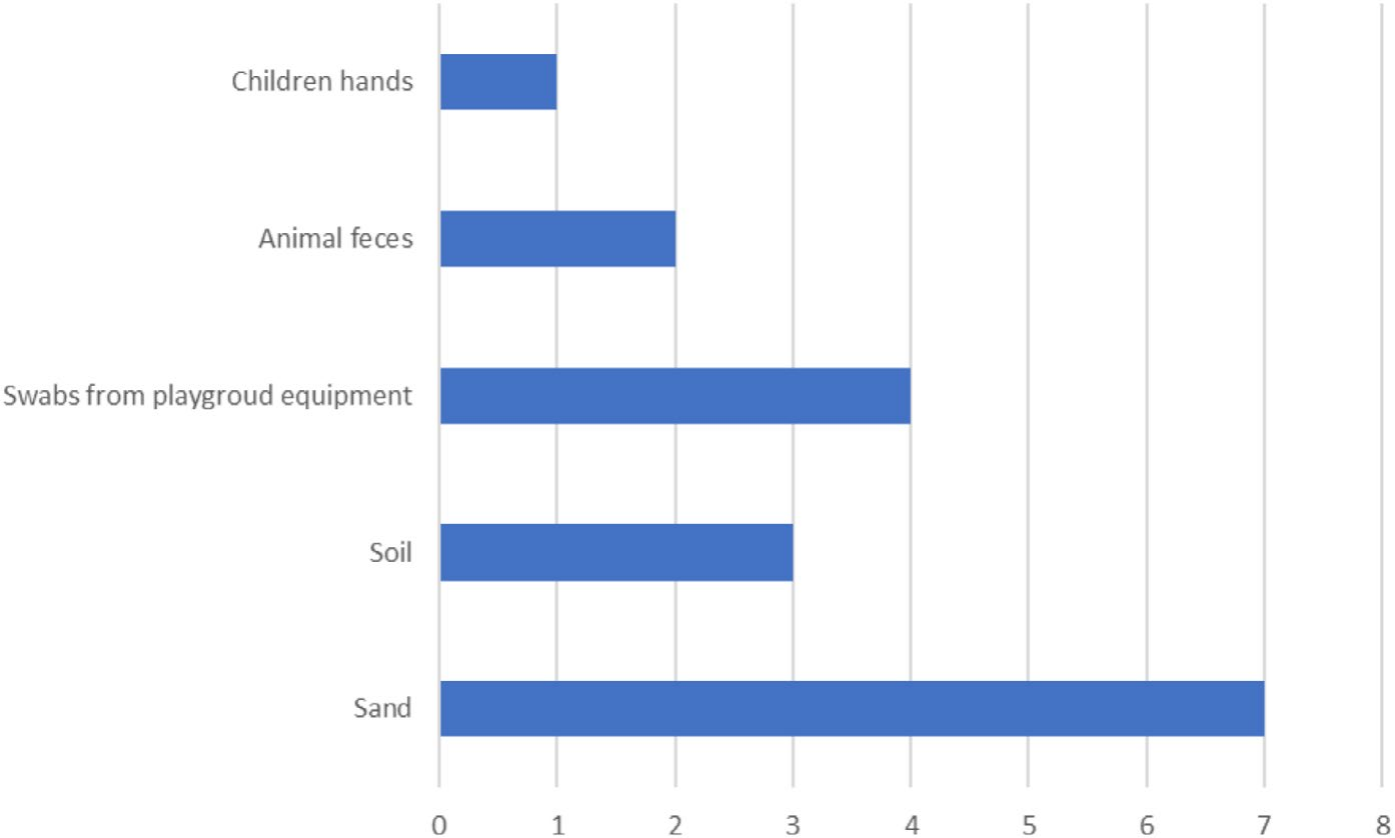
Number of publications reporting data on playground‐associated microbes, classified by sample origin. Data are based on (Table 3).

Most studies focus on analysing only selected sites within the playground areas, while there is a significant lack of research that comprehensively maps the distribution of microorganisms across entire playgrounds. The most commonly analysed samples are sand from sandboxes (Badura et al. 2014; Zatoń and Błaszak 2015; Orden et al. 2017; Chatziprodromidou et al. 2021) or soil (Haddock and Nocon 1986; Pérez et al. 2010; Caliskan et al. 2021). Less frequently, swabs are taken from the surfaces of playground equipment (Thapaliya et al. 2019; Ababneh et al. 2022; Caliskan et al. 2021). Very rarely, other types of samples are examined, such as those collected directly from the bodies of playground users (e.g., children's hands) (Simanjuntak et al. 2023) or faecal samples from animals (wild birds and canines) found on playgrounds (Abdollahpour et al. 2015; Ortega‐Paredes et al. 2019) (Table 3 and Figure 3).

### 3.2 Laboratory Procedures

The collected samples were typically analysed using standard laboratory procedures commonly employed in microbiology. These procedures included culturing bacteria isolated from the environment, plating them on selected microbiological media, isolating single colonies, conducting species identification and testing for antimicrobial susceptibility.

A common procedure involved using enrichment culturing, which consists of pre‐cultivating the collected samples on nutrient‐rich standard growth media, followed by inoculating the obtained bacteria on appropriate selective media (Badura et al. 2014; Caliskan et al. 2021; Ababneh et al. 2022). Another frequently used approach was to inoculate the collected samples directly onto appropriate selective media, such as *Burkholderia cepacia* selective agar for *Burkholderia cepacia* (Miller et al. 2002) or CHROMagar Staph aureus for *Staphylococcus aureus* (Horváth et al. 2024). If isolation of single bacterial colonies was not required, culturing was omitted, and the organism was identified directly from collected samples by real‐time PCR assays. This is the method used to identify *Helicobacter pylori* (Pérez et al. 2010), *Chlamydophila* spp. and *Salmonella* spp. (Tarsitano et al. 2010). Studies that included preliminary bacterial isolation onto appropriate media often used PCR‐based methods for molecular identification (Miller et al. 2002; Thapaliya et al. 2019; Chen et al. 2020; Ababneh et al. 2022; Horváth et al. 2024). In more recent reports, MALDI‐TOF‐based procedures were increasingly used (Badura et al. 2014; Ortega‐Paredes et al. 2019; Caliskan et al. 2021; Simanjuntak et al. 2023), whereas older studies sometimes relied on biochemical test kits for bacterial identification (Davis et al. 1999; Ayan 2013).

Antibiotic susceptibility testing was usually performed using the disk diffusion method (Badura et al. 2014; Ortega‐Paredes et al. 2019; Caliskan et al. 2021; Ababneh et al. 2022) or the VITEK 2 automated system (BioMérieux) (Badura et al. 2014; Thapaliya et al. 2019; Chatziprodromidou et al. 2021). Other techniques, such as the agar dilution method or those involving ETEST strips (BioMerieux), were used less frequently (Orden et al. 2017; Horváth et al. 2024).

### 3.3 The Most Commonly Studied Bacterial Taxa

Most studies focus on confirming specific bacterial taxa, estimating contamination frequency, or measuring bacterial load. The representativeness of these results depends largely on sample size, which was substantial ‐ reaching hundreds of repetitions ‐ in only a few studies (e.g., Caliskan et al. 2021) (Table 3).

The most frequently studied bacteria were potentially pathogenic species that could pose health risks to playground users (Table 3). These bacteria included (in alphabetical order): *Acinetobacter pittii, Campylobacter* spp., *Clostridium difficile, Enterococcus faecalis, Escherichia coli, Helicobacter pylori, Pseudomonas aeruginosa, Salmonella* spp., and *Staphylococcus aureus*.

*Acinetobacter pittii* is not considered as highly pathogenic as the closely related, carbapenem‐resistant strains of *A. baumannii* . Nevertheless, *A. pittii* is increasingly associated with several types of severe hospital infections and is becoming an emergent pathogen of concern (Bello‐López et al. 2024). The emergence of carbapenem resistance in *A. pittii* is particularly worrisome.

*Campylobacter* spp. (primarily *C. jejuni* ) and *Clostridium difficile* cause gastrointestinal diseases that can lead to serious diarrheal infections. *Campylobacter* infections are one of the two most common causes of foodborne illness in the United States, with children under 5 years of age being particularly at risk. The most common illness caused by *C. jejuni* is gastroenteritis, which can lead to severe complications, including reactive arthritis and Guillain‐Barré syndrome (Same and Tamma 2018).

*C. difficile* is the pathogen most commonly responsible for healthcare‐associated diarrhoea. This organism can colonise the large intestine under specific conditions (e.g., after broad‐spectrum antibiotic therapy), causing varying degrees of damage, from mild diarrhoea to toxic megacolon (Dop et al. 2023). A particularly problematic feature of *C. difficile* is its ability to produce heat‐resistant and aero‐tolerant spores, which allows it to persist on surfaces for several months (Rexach et al. 2006). This pathogenic trait significantly increases the risk of infection in public places such as playgrounds.

*Enterococcus faecalis* is a natural inhabitant of the human gut microbiota, but when immune suppression occurs, it can cause a variety of pathologies, including urinary tract infections, endocarditis, septicemia, and wound infections. Because *E. faecalis* develops resistance to many common antibiotics, treating these diseases is often challenging (Singh et al. 2019).

Although *Escherichia coli* is a common constituent of the human gastrointestinal microbiota, pathogenic variants of this organism can cause various diseases, including diarrhoea, urinary tract infections, sepsis, and meningitis (Kaper et al. 2004). Diarrheal disease caused by pathogenic *E. coli* variants leads to high mortality, especially among children under 5 years of age in sub‐Saharan African and South Asian countries (Croxen et al. 2013).

*Helicobacter pylori* is a bacterium that colonises the gastric mucosa and, although often asymptomatic, is the leading cause of peptic ulcers and gastritis worldwide. Persistent colonisation of *H. pylori* in adults increases the risk of stomach cancer, while early infections of this pathogen in children are associated with nodular gastritis (Mehrabani 2019).

*Pseudomonas aeruginosa* is an opportunistic pathogen associated with severe infections, including pneumonia, urinary tract infections, wound infections, skin and soft tissue infections, and ear infections. These bacteria are among the most important etiological agents of pneumonia in children under 12 years of age. Due to multiple complementary antibiotic resistance mechanisms, they cause high morbidity and mortality (Chen et al. 2018; Sathe et al. 2023).

*Salmonella* species cause foodborne illnesses manifested by gastroenteritis with diarrhoea, abdominal pain, and fever. The main representative is *S. enterica* , a species comprising more than 2600 serotypes, including those causing typhoid fever or paratyphoid fever (Chen et al. 2023).

*Staphylococcus aureus* causes a wide range of infections, from skin abscesses and endocarditis to respiratory infections and severe bloodstream infections. Methicillin‐resistant strains pose a particular threat due to their resistance to standard antibiotics (Tong et al. 2015). These bacteria are among the 15 antibiotic‐resistant species listed by the World Health Organization (WHO 2024) as causative agents of acute infections that pose high risks of mortality and morbidity.

### 3.4 Analysis of Antibiotic Resistance in Bacteria Collected From Playgrounds

The antibiotic resistance profile of bacteria isolated from playgrounds has been rarely studied. Table 4 summarises data from publications that have addressed this issue. The studies are compared according to the antibiotics used and the level of susceptibility of the bacteria tested. Notably, susceptibility to each specific antibiotic varied significantly depending on the location of the study. In this respect, it would be interesting to investigate a possible correlation between antibiotic use in each particular country and the level of resistance to that agent displayed by bacterial isolates. The most frequently tested isolates, *E. coli* , exhibited resistance to ampicillin (12%–22%), ciprofloxacin (0%–8.7%), gentamycin (0%–8.7%), streptomycin (8.7%–17.6%), and trimethoprim/sulfamethoxazole (5.8%–21.8%). No resistance was detected to amikacin, aztreonam, cefepime, cefotaxime, ceftazidime, ertapenem, fosfomycin, imipenem, meropenem, or tigecycline (Table 4). In the case of *S. aureus* , the highest prevalence of MRSA was found in swabs from crawl tunnels (15%), spring riders (11.1%), and slide edges (10.5%) (Thapaliya et al. 2019). Strains isolated from playground elements also showed resistance to ciprofloxacin, clindamycin, erythromycin, and penicillin (Horváth et al. 2024) (Table 4).

## 4 Factors Influencing Microbiological Safety in Playgrounds

Studies indicate that playgrounds can harbour potentially pathogenic bacteria, some of which may exhibit varying degrees of antibiotic resistance. It is reasonable to assume that, even if the overall level of bacterial contamination in playgrounds remains constant at current levels, the proportion of antibiotic‐resistant strains will continue to increase. This is due to the growing prevalence of antimicrobial resistance in the environment, with various sources and transmission vectors being continually identified. In light of this, the following sections examine the factors that may contribute to the elevated presence of bacteria on playgrounds, thereby impacting the microbiological safety of playground users.

### 4.1 Influence of Material Type and Surface Characteristics on Microbial Contamination

The selection of construction materials is a critical factor influencing the microbiological safety of playgrounds, as bacterial survival and affinity for different materials vary widely and depend primarily on surface energy and charge, topography, and wettability (Wilks et al. 2005; Tomičić et al. 2020; Iyer et al. 2023). For examples, surfaces with moderate wettability, rough topography, and higher surface energy are more prone to bacterial attachment and cell accumulation, whereas smoother and less porous materials generally support lower microbial loads (Yuan et al. 2017).

Although loosely attached microbes are readily removed by shear forces and swept away, they become tightly bound to the surface through the secretion of extracellular polymeric substances (mainly exopolysaccharides). The subsequent formation of a microbial biofilm ensures an irreversible attachment to the material surface (Cookson et al. 2002). Biofilm composition varies among microbial species, and the abiotic surface properties may trigger different colonisation mechanisms (Iyer et al. 2023). Many biofilms are sufficiently thick to be visible to the naked eye, but their formation on the playground equipment and surfaces could lead to disease transmission. Furthermore, biofilms are challenging to remove and exhibit increased resistance to antimicrobial agents compared with planktonic bacteria. The application of surfacing and materials with antibacterial properties or poor bacterial adherence characteristics could help prevent cross‐contamination events during play if standard hygiene practices fail.

Different construction materials commonly used in playgrounds ‐ such as metals, plastics, and wood ‐ differ considerably in their susceptibility to microbial colonisation, biofilm formation, and long‐term pathogen survival. A brief overview of these materials in the context of microbiological safety is presented below.

#### 4.1.1 Metals

Stainless steel ‐ either coated or painted ‐ is commonly used for slide surfaces, fasteners, and structural components. However, *E. coli* and *Salmonella* spp. can adhere to both stainless steel, and *E. coli* is capable of surviving on stainless steel for over 28 days at both 20°C and 4°C (Merritt et al. 2000; Carvalho et al. 2023; Wilks et al. 2005). While stainless steel offers excellent mechanical strength and corrosion resistance, it lacks intrinsic antimicrobial properties. In contrast, copper possess antibacterial properties against various microorganisms, including *E. coli* and MRSA (Wilks et al. 2005; Salah et al. 2021). However, it exhibits poor corrosion resistance and limited durability making it unsuitable as a surface material for outdoor applications (Wilks et al. 2005). Copper‐containing alloys (such as brass, bronze, copper‐nickel, and copper‐silver alloys) also demonstrate antimicrobial activity (depending on their copper content) and are more suitable for general use due to enhanced durability and corrosion resistance (Salah et al. 2021).

#### 4.1.2 Plastics

UV‐stabilised high‐density polyethylene (HDPE) and rotationally moulded low‐density polyethylene (LDPE) are widely employed for activity panels, signs, slides, and tubes due to their durability, resistance to corrosion, low maintenance requirements, and favourable aesthetic properties. Nevertheless, these polyethylene surfaces are also prone to microbial contamination (Merritt et al. 2000; Carvalho et al. 2023; Wilks et al. 2005).

#### 4.1.3 Wood

It is considered an environmentally friendly material commonly used in city parks and playgrounds. Bacteria such as *E. coli* , *P. aeruginosa*, and *S. aureus* can adhere to wooden surfaces, but differences have been observed depending on the test strain and type of wood (Tomičić et al. 2020). It was also shown that wood treated with a combination of oil (bioimpregnation agent) and a disinfectant enhances the adhesion of *P. aeruginosa* on the beech surfaces while inhibiting the attachment of *E. coli* and *S. aureus* (Tomičić et al. 2020). Notably, some wood types exhibit antibacterial and antifungal properties, likely due to their chemical composition (Vainio‐kaila et al. 2015). For example, pine extracts have demonstrated strong antibacterial effects against methicillin‐resistant *Staphylococcus aureus* (MRSA), vancomycin‐resistant *Enterococcus faecalis* (VRE), and *Streptococcus pneumoniae* but weaker on *E. coli* (Vainio‐kaila et al. 2015). Thus, the application of this type of wood in a playground environment may offer additional hygienic benefits.

### 4.2 Influence of Green Spaces and Wildlife on Playground Microbiological Safety

Urban green spaces provide opportunities for socialisation and have a positive effect on the well‐being of their visitors (Ayala‐Azcárraga et al. 2019). This is likely due to the presence of trees and natural sounds, which contribute to stress reduction and improved mood, for example, by creating a pleasant environment (Ayala‐Azcárraga et al. 2019). Access to nature improves children's psychological well‐being and encourages environmental stewardship (Reedy 2024). The presence of natural elements in playgrounds also encourages children to develop risk management skills and adapt their behaviour to the surroundings (Reedy 2024). Consequently, any feature that promotes contact with nature ‐ such as environmental elements, natural shapes, and forms ‐ is becoming increasingly popular in modern playground design (Russo and Andreucci 2023). Maintaining natural substrates is also important, as replacing natural soil with artificial materials (rubber mats) alters the microbial balance of play environments (Manninen et al. 2025).

Conversely, green areas harbour greater animal diversity (e.g., pigeons, rats, mice, squirrels, or dogs and cats), which may serve as reservoirs or transmitters of diseases, not to mention the pet owners who may also act as vectors of zoonotic illnesses such as skin and nail fungal infections, diarrhoea, salmonellosis, toxocarosis and toxoplasmosis (Haddock and Nocon 1986; Abdollahpour et al. 2015; Otero et al. 2018; Pacheco‐Ortega et al. 2019). Additionally, the presence of dense vegetation may favour the accumulation of garbage on the site (Ayala‐Azcárraga et al. 2019), whereas contamination of sand in playground areas or sandpits with animal faeces or dead animals increases the risk of infection with parasites (Otero et al. 2018; Pacheco‐Ortega et al. 2019) and *Salmonella*, respectively (Haddock and Nocon 1986).

Furthermore, multidrug‐resistant *E. coli* were isolated from animal faeces collected from playground zones (Ortega‐Paredes et al. 2019). Thus, enclosing the playground with fencing is generally required to protect it from animals (Błaszak and Zatoń 2015). Food waste and garbage, however, attract animals and promote bacterial and fungal growth, as they provide organic compounds for these microorganisms (Błaszak and Zatoń 2015; Zatoń and Błaszak 2015). Despite installing fences and nets, certain animals (e.g., cats, birds, dogs) still have occasional access to the playground area. However, these physical barriers reduce pollution derived from waste and plant material in the sandpits (Błaszak and Zatoń 2015). Interestingly, studies on the microbiological safety of sandpits revealed that the presence of fencing, shading, nearby aquatic habitats and contamination with waste or animal faeces does not fully account for the presence of *E. coli* and coliform bacteria in sand samples (Błaszak and Zatoń 2015; Caliskan et al. 2021). However, in unfenced sandpits, a significantly higher number of bacteria and fungi were detected compared with the fenced ones (Błaszak and Zatoń 2015).

### 4.3 Influence of Ambient Conditions and Environmental Factors on Microbial Survival

Ambient conditions influence the survival of microbiomes outside their host environment. Faecal bacteria such as *Enterococcus* spp. and *E. coli* exhibit varying abilities to cope with environmental stressors such as large fluctuations in temperature and pH, salinity, sunlight, and limited nutrient availability (Halliday and Gast 2011). Sudden changes in moisture are stressful for microorganisms, which must expend energy to regulate osmotic pressure in their microenvironment (Scoullos et al. 2019). It was also demonstrated that exposure to light promotes the die‐off of *E. coli* on the surface of dark‐coloured urban materials (such as rubber and asphalt) and sand due to temperature increases (Scoullos et al. 2019). In contrast, UV irradiation for up to 6 min. does not destroy *E. coli* in a 30 cm deep sand plot (Beversdorf et al. 2007). Furthermore, this organism can survive for several weeks in the sand, especially at lower temperatures (15°C) (Staley et al. 2016). These observations are in line with data reporting a lower survival rate of *E. coli* at higher temperatures (Scoullos et al. 2019).

It has also been documented that faecal bacteria persist longer in wet sand than in water (Halliday and Gast 2011). Furthermore, the number of bacteria and fungi in sand may vary widely throughout the year and depend on climate conditions. In Greece, a higher number of *E. coli* , *P. aeruginosa* , and *S. aureus* in soil collected in the playground zone was reported during the wet season (temperature about 9°C) compared with the dry one (temperature about 48°C) (Chatziprodromidou et al. 2021). In contrast, in Poland (Central Europe), a similar content of bacteria and fungi was detected both in spring and autumn in sand from fenced sandpits. On the other hand, monitoring of unfenced sandpits revealed higher contamination levels with bacteria and fungi in autumn compared with spring (Błaszak and Zatoń 2015). In line with this observation, higher bacterial and fungal loads were determined in autumn than in springtime in sand collected from the playground zone of Polish artificial beaches (Zatoń and Błaszak 2015). Finally, factors such as air temperature and water content appear not to influence the concentration of *E. coli* in sand, whereas sand's water content affects coliform bacteria's content (Zatoń and Błaszak 2015).

Concerning microbial adhesion, both humidity and temperature significantly affect the ability of microorganisms such as *P. aeruginosa* , *E. coli* , *S. aureus* , and the yeast *Pichia membranifaciens* to adhere to wooden surfaces (Tomičić et al. 2020). Notably, the highest degree of adhesion to this biomaterial was observed at a relative humidity of 98% and temperatures ranging from 20°C to 37°C for bacteria, or from 20°C to 27°C for yeast (Tomičić et al. 2020).

### 4.4 Operational Factors ‐ Children's Age‐Related Behaviour and Play Preferences

When playing in public areas, children acquire microbial contamination from soil, toys, and other fomites, as well as from their playmates. Children themselves contribute to the contamination of playground equipment and surfaces with their own microbiota, transferring microorganisms from the soles of their shoes, hands, and other body parts to their surroundings (Błaszak and Zatoń 2015). Furthermore, during play, opportunistic and pathogenic microorganisms are transferred from soil to children's hands and toys and vice versa (Martínez‐Bastidas et al. 2014). Thus, surfaces frequently touched by children's hands should be considered important transmission points of resistant bacteria between humans.

Undoubtedly, children's age plays an important role when evaluating risk factors for exposure to microbial pathogens. In fact, children engage in age‐specific activities in playgrounds (Simanjuntak et al. 2023). Specifically, children aged 1–10 years are more prone to exposure to microbial pathogens (e.g., bacteria causing diarrhoea and/or nosocomial infections), as they tend to explore and seek new experiences. Furthermore, toddlers require special attention due to their high activity levels and oral exploratory behaviour, which increases the likelihood of ingesting contaminants (including soil and faeces) during crawling (Simanjuntak et al. 2023; Cantrell et al. 2023). For example, a study conducted in Greece showed that children aged 6–9 have a higher risk of infection by certain pathogens ‐ *P. aeruginosa* > *E. coli* > *S. aureus* ‐ when playing in the playground for 1 h each day (Chatziprodromidou et al. 2021).

Importantly, children's preferences for certain playground materials also influence their exposure to microbes. Survey data indicate that children aged 5–12 years favour plastic elements, followed by wood rather than metal, in play zones (Kus Sahin and Onay 2020). As a result, surfaces made of plastic and wood are more frequently touched and contacted, potentially increasing the likelihood of microbial contamination and transmission.

Hands are important intermediaries in the transmission of faecal indicator bacteria as well as enteric and opportunistic pathogens such as *E. coli* , faecal coliforms, enterococci, adenovirus, and norovirus (Cantrell et al. 2023). The presence of *Giardia lamblia* cysts, faecal coliforms, *E. coli* , *S. aureus* , *Klebsiella pneumoniae* , *Salmonella* spp., and *Serratia* spp. has been detected on the hands and toys of children playing in parks and/or on sidewalks (Martínez‐Bastidas et al. 2014). In another study, the highest burden of hand contamination with Gram‐negative bacteria was observed in children aged 1–4 and 5–9 years, compared with younger (< 1 year) and older children (10–14 years) (Simanjuntak et al. 2023). In this study, *Pseudomonas* species were the predominant organisms. It was also demonstrated that *E. coli* strains isolated from swab samples of different playground equipment (slides, swings, ferris wheels, seesaws) exhibited higher resistance rates to antibiotics such as ampicillin, trimethoprim‐sulfamethoxazole, and streptomycin than isolates obtained from soil samples (Caliskan et al. 2021).

### 4.5 Hand Hygiene and Education

Education, living conditions, and hygiene‐related practices are examples of factors influencing children's exposure to faecal bacteria (Simanjuntak et al. 2023). There is a higher prevalence of hand contamination with *E. coli* and faecal coliforms in low‐income countries than in high‐income countries (Cantrell et al. 2023), which can be at least partially attributed to differences in access to handwashing facilities. Interestingly, no significant differences in the prevalence of *E. coli* and faecal coliforms between the hands of children under 5 years old and adult hands have been reported, suggesting similar hand hygiene practices in adults and children (Cantrell et al. 2023).

## 5 Strategies for Improving Microbiological Safety in Playgrounds

Determination of new strategies against antibiotic resistance requires intersectoral collaboration and efforts encompassing all possible opportunities for science and technology. Cleaning with water, soap (or a neutral detergent), and some form of mechanical action (brushing or scrubbing) removes and reduces dirt, debris, and other organic matter, but does not necessarily eliminate microorganisms (WHO 2020). Classical methods such as chemical disinfection and sterilisation are used to remove pathogens, but environmental protection calls for alternative approaches (Isopencu and Mocanu 2022). Thus, the development of antibacterial materials for personal protection and environmental security has rapidly progressed to address the problem of the spread of multidrug‐resistant bacteria (Luo et al. 2021). These materials are designed either to exhibit bactericidal activity (killing microorganisms) or to display bacteriostatic properties (preventing their attachment, survival, growth, and biofilm formation) (Mahanta et al. 2021).

According to available standards (Table 2), materials with good shock‐absorbing capacity, low toxicity, and a suitable level of protection against corrosion and insect intrusion are preferable for playground applications. Although it is well documented that bacterial accumulation onto surfaces occurs in almost any environment (Cookson et al. 2002), recommendations for selecting materials with good antibacterial properties or low affinity for microbial attachment and biofilm formation have not been included in any legal norm.

One of the current trends is the fabrication of antibacterial polymeric materials by combining various microbicidal agents such as N‐halamine, antibiotics, nitric oxide, natural essential oils, and quaternary ammonium compounds (Luo et al. 2021). These materials can be produced using different synthesis strategies and then manufactured into products such as nanoparticles, fibres, membranes, or coatings (Luo et al. 2021). In particular, antibacterial coatings have been applied to various substrates, including stainless steel, to prevent bacterial colonisation (Luo et al. 2021) and might be useful for outdoor applications. Furthermore, the organic biocides could be replaced by nanomaterials with antimicrobial activity such as silver, titanium dioxide and zinc oxide (Ganguli and Chaudhuri 2021). For instance, silver nanoparticles have been shown to inhibit or reduce microbially induced degradation of household paints, gypsum, grout and other outdoor assets (da Silva et al. 2019; Ganguli and Chaudhuri 2021).

Despite the ability of biocidal polymers to inactivate bacteria, the accumulation of debris from dead microorganisms can facilitate secondary contaminations. Therefore, materials with antifouling properties are also desirable for playground surfacing (Luo et al. 2021). Among these, antifouling paints ‐ commonly used to prevent the attachment and growth of marine organisms on ship and boat hulls ‐ typically contain copper or zinc as biocides (Lagerström et al. 2020). However, leaching of these biocides from coating to the environment is a real threat (Lagerström et al. 2020). Furthermore, there is concern that not only antibiotics but also heavy metals and biocides contribute to the development of antibiotic resistance through co‐selection mechanisms (Flach et al. 2017). For example, biofilm bacteria collected from plastic panels painted with a copper and zinc‐containing antifouling pigment have shown increased resistance to both heavy metals and tetracycline (Flach et al. 2017).

While advances in the development of new construction and surface materials for playgrounds contribute to improved microbiological safety, these technological solutions should be complemented by effective hygiene practices and targeted educational interventions. Practicing effective hand‐washing techniques by children might significantly reduce the risk of diarrheal disease in children (Cantrell et al. 2023). Moreover, cleaning and disinfecting toys and playground equipment might be crucial to reducing microbial dispersion. Many non‐hazardous and non‐toxic products (also with biodegradable ingredients) effective for cleaning or disinfecting surfaces (including children's toys) are available commercially (Jimenez et al. 2010). Detergents with enzymes and sodium hypochlorite (NaOCl) have also been found to be effective at removing pathogenic organisms from contaminated surfaces (Cookson et al. 2002).

## 6 Conclusions

A systematic literature review revealed that targeted empirical studies on the presence of pathogenic bacteria in playgrounds have been conducted relatively infrequently and in only a few countries worldwide. Even less frequently has the issue of antibiotic resistance in playground‐isolated bacteria been addressed. We highlight significant gaps in knowledge regarding this topic and the lack of dedicated legal regulations governing microbiological safety on playgrounds.

However, existing data clearly indicate that pathogenic bacteria, from various species, are present in areas where children play and that these bacteria can exhibit diverse antibiotic resistance traits. Given that antibiotic‐resistant strains represent an increasingly serious global public health issue, there is a need to develop global strategies to better protect playgrounds from resistant pathogens. We propose that the most common and globally applicable approach would be the introduction and use of appropriate materials with antibacterial properties. This recommendation stems, in part, from the fact that various environmental factors responsible for increasing bacterial loads cannot always be fully controlled. Finally, we also emphasize the importance of hygiene practices and public education regarding existing risks, without discouraging the use of playgrounds by children.

## Author Contributions

**Rafał Łopucki:** conceptualization, investigation, writing – original draft, writing – review and editing, data curation, formal analysis, funding acquisition. **Marcin Skowronek:** writing – original draft, writing – review and editing, formal analysis. **Anna Bilokinna:** writing – review and editing, formal analysis. **Guillermo Martinez‐de‐Tejada:** writing – review and editing, formal analysis. **Ilona Sadok:** investigation, writing – original draft, writing – review and editing, formal analysis, data curation, funding acquisition.

## Conflicts of Interest

The authors declare no conflicts of interest.

## Data Availability

The dataset analysed during the current study is available from the corresponding author on reasonable request.
